# Indirect Magnetic Resonance Imaging Lymphography Identifies Lymph Node Metastasis in Rabbit Pyriform Sinus VX2 Carcinoma Using Ultra-Small Super-Paramagnetic Iron Oxide

**DOI:** 10.1371/journal.pone.0094876

**Published:** 2014-04-14

**Authors:** Na Shen, Jun Tan, Peng Wang, Jian Wang, Yuan Shi, Wenqi Lv, Xiaofeng Xie, Xinsheng Huang

**Affiliations:** 1 Department of Otolaryngology, ZhongShan Hospital, Fudan University, Shanghai, China; 2 Department of Radiology, ZhongShan Hospital, Fudan University, Shanghai, China; 3 Department of Pathology, ZhongShan Hospital, Fudan University, Shanghai, China; 4 Department of Polymer Science, Fudan University, Shanghai, China; Health Canada and University of Ottawa, Canada

## Abstract

**Background:**

USPIO is a contrast agent for MRI that can generate T2W images with low signal intensities. After subcutaneous or intravenous injection of USPIO, normal lymph node tissues uptake these nano-particles, but tumor cells do not. Thus, tumor metastasis can be detected using this contrast agent.

**Objective:**

The aim of this study was to access the feasibility of USPIO enhanced MRI for the detection of cervical lymph node metastasis in a pyriform sinus carcinoma animal model and to investigate the ability of USPIO to enhance images of cervical lymph node metastases.

**Methods and Findings:**

Twenty New Zealand rabbits were randomly divided into tumor and inflammatory groups, and each group contained 10 rabbits. In the inflammatory group, a 0.5 ml egg yolk emulsion was injected into the sub-mandibular muscle of the rabbits to induce an inflammatory reaction in their cervical lymph nodes. In the tumor group, a VX2 tumor tissue suspension was transplanted into the pyriform sinus sub-mucosa of the rabbits using direct laryngoscope. Four weeks after the tumor or egg yolk injection, MRIs were performed before and after USPIO injection to observe the imaging enhancement features of USPIO. After that, a histo-pathological analysis was performed for all rabbits. We found the metastatic lymph nodes had no signal reduced intensity or irregular signal reduced intensity on T2-weighted image by using USPIO enhancement. In the tumor group,the sensitivity and specificity of plain MRI were 57.6% and 60.7%. The corresponding values of USPIO-enhanced MRl were 96.1% and 85.7%. (P<0.05)

**Conclusion:**

The features and the extent of the lymph node metastases corresponded to those observed on USPIO-enhanced MR images. USPIO-enhanced MRI is useful for the detection and estimation of lymph node metastasis in this cervical carcinoma animal model.

## Introduction

Pyriform sinus carcinoma is one type of the hypo-pharyngeal carcinoma. As it is poorly differentiated and difficult to diagnose early, lymph node metastases are frequently found in patients and result in a poor patient prognosis [1]. Thus, the early diagnosis of lymph node metastases is important in the clinical [2], [3]. There are many methods of detecting lymph node metastases, such as B ultrasound, CT, MRI and PET. B ultrasound, CT and MRI demonstrate the size rather than the architecture of tumors, but with those imaging methods, it is occasionally not possible to determine whether a lymphatic metastasis will develop when the lymph node has a normal size [4]. PET is an advanced imaging technique with high accuracy, but it has poor spatial resolution and structure development [5]. Therefore, a new imaging technique that allows for an accurate preoperative assessment of the neck lymph nodes would be valuable. It has been reported that lymphography has the unique capability of visualizing the internal architectural derangements of even normal-sized lymph nodes [4]. There are two types of lymphography: direct and indirect. Direct lymphography is complicated because it requires the dissection of the lymph-vessel; this limits its application. Indirect lymphography is more commonly used with CT and MRI. After injecting contrast agents into the interstitial fluid, they pass to the lymph-vessel through diffusion or phagocytosis, and then the lymph node is imaged. There are no lymph-vessels in tumor tissues, so the contrast agent filling defected would appear on metastatic nodes. Thus, the interstitial delivery of diagnostic agents has been useful for detecting lymph node metastases and assessing lymphatic function either by indirect computed tomography lymphography or indirect magnetic resonance image lymphography [6].

The paramagnetic material was made directly into nano-particles for use as MRI indirect lymphatic contrast agents. According to size, the contrast agents were divided into two types: super-paramagnetic iron oxide (SPIO, size >100 nm) and ultra-small super-paramagnetic iron oxide (USPIO, size <  = 20 nm). Through phagocytosis by the reticulo-endothelial system, super-paramagnetic iron oxide becomes mainly distributed in the liver and spleen [7]. The contrast agent used in this study was the ultra-small super-paramagnetic iron oxide; after intravenous administration, only a few nano-particles were absorbed by the liver and spleen, while most were distributed to the lymph nodes in all parts of the body. Thus, USPIO could be considered a targeted contrast agent for the lymph node. In this study, we assumed that USPIO could distribute throughout the body's lymphatic system, local concentrations would be low, and the surrounding blood vessels would be revealed by imaging, and the specificity would also be low. Thus, we used a sub-mucosal injection around the tumor site to achieve regional lymph node drainage [8], [9]. USPIO is a newly negative MRI contrast agent. After subcutaneous or intravenous injection, normal lymph node tissue can absorb USPIO particles, but metastatic lymph nodes can only absorb little to no USPIO particles as their phagocytes are damaged or their lymphatic drainage is blocked by tumor cells. The signal intensity of normal lymph nodes in SE T2WI and SE GRET2WI decreased obviously, while that of metastatic lymph nodes only changed minimally or not at all. Recently, other studies into the diagnosis of lymph node metastases have been reported for breast, gastric and prostate cancer [10]–[12]. Those works provided evidence for our research. In this study, the MRI visualization of lymph node metastases of rabbit VX2 pyriform sinus carcinomas can be enhanced by MR scanning after injecting USPIO into the sub-mucosa around the tumor prior to performing the MRI.

## Material and Methods

### 1. Animal model

Fifteen New Zealand white rabbits, weighing 2.0 to 3.0 kg, were bred and provided to us by the Laboratory Animal Center of Shanghai Public Health Clinic Center under routine conditions, according to the institute's ethical and environmental guidelines. The rabbits were divided into two groups, with 10 rabbits each group, by a random number table method. The ethics committee of the Shanghai Public Health Clinic Center approved this study.

#### 1.1 Preparation of VX2 tumor mass suspension

The tumor cells had been continuously passed in rabbits intramuscularly. The rabbits were anesthetized, and the tumor cells were then implanted into the rabbits' thigh muscles. After the tumor grew to a palpable size, the rabbit was sacrificed using an intravenous overdose of pentobarbital. The tumor was then excised. Muscle and necrotic tissue were removed. The tumor was placed in a petri dish with saline. Four 1-cm^3^ pieces were selected and cut with eye scissors into pieces no bigger than 1 mm^3^. These pieces were then placed in another dish with 40 ml balanced saline and were suspended at a concentration of 100 pieces per ml.

#### 1.2 Tumor implantation

Tumor implantation was performed under general anesthesia. The rabbits were premedicated with 60 mg/kg of 5% ketamine injected intramuscularly. After 5 minutes, a “butterfly” needle was inserted into the marginal vein of the ear, and 15 mg/kg pentobarbital sodium was injected slowly. The animals breathed without the aid of a respirator. Anesthesia was maintained up to 30 minutes with good analgesia and muscle relaxation. The anesthetized rabbits were placed in a supine position on a specially designed operating table that allowed for the extension of the neck. A pediatric direct laryngoscope was used to expose the pyriform sinus. The VX2 mass suspension was drawn up into a 1 ml syringe with a retropharyngeal puncture needle (1.2 mm diameter) and 0.5 ml were injected into the submucosa of the lateral wall of the pyriform sinus. Injection into the site was confirmed by visualization of a small mucosal bleb.

#### 1.3 Inflammatory model establishment

Fresh eggs mixed were with normal saline at a proportion of 10%, and an egg yolk emulsion was made from the complete oscillation of this mixture. Then, the egg yolk emulsion was injected into the sub-mandibular region of the rabbit. For two weeks, this procedure was repeated every three to four days later.

### 2. Magnetic resonance imaging

Four weeks after the tumor or egg yolk injection, an MRI scan was conducted. All MRI examinations were performed with a 1.5-T MRI unit (Siemens, Shanghai, China). Ten nano-particles of negatively charged USPIO was used, and contained 1.9 mg per Fe milliliter (Beijing WanDe New Technology Co., Ltd.). The USPIO was diluted with 1 mg Fe per kilogram. After anesthesia, the MRI scan was measured. We then injected the USPIO solution into the submucosa of the pyriform sinus around the tumor. Twelve hours later, the USPIO enhancement scan was conducted. The MRI imaging parameters were as follows: T2WI (TR/TE 2400/7.6 ms, field of view (FOV) 18×18 cm, matrix 256×256, section thickness of 3 mm, and 4 excitations.) and T1WI (TR/TE 400/12 ms, field of view (FOV) 18×18 cm, matrix 256×256, section thickness of 3 mm, and 4 excitations).

### 3. Image analysis

Two experienced radiologists analyzed the image data. (J.W and JZ.L) The images were randomly viewed using a picture archiving and communication system workstation. The two radiologists were blind to each other's radiologic evaluation results and to the histo-pathologic results. In cases of discrepancies between the two readers, a consensus was reached through discussion. Detected nodes were classified into three patterns [13]: Mode A: T1WI and T2WI were low signals, judged to be normal lymph node; Mode B1: T1WI and T2WI both had part of high signals in some areas, judged to be normal lymph node; Mode B2: T2WI had some high signal areas, while T1WI had only low signal areas, judged to be metastatic lymph nodes. The signal intensity (SI) on T2WI was measured before and after USPIO enhancement using regions of interest (ROI). (T2_SI_  =  (SI_PRE_-SI_POST_)/SI_POST_×100%)

### 4. Histological evaluation

The rabbits were sacrificed using an intravenous overdose of pentobarbital after the MRI scan. Afterwards, a complete cervical lymph node dissection was performed and the rabbit's pyriform sinus was observed and excised. The locations and sizes of the dissected lymph nodes were recorded for all rabbits. Then, the dissected lymph nodes and pyriform sinus tumors were fixed in 10% formalin in saline for at least 24 hours. The sections were stained with hematoxylin and eosin (HE).

### 5. Statistical analysis

SPSS 18.0 software was used for the data analysis. We compared the MRI results with the pathological results, and calculated the sensitivity and specificity of the USPIO enhancement scan. We compared the positive rate of the USPIO enhancement scan and MRI scan using a chi-square test. We compared the T2 value before and after injection using a paired t-test. (P<0.05)

## Results

### 1. Tumor-bearing group

#### 1.1 Histopathologic results

**Figure 1:**
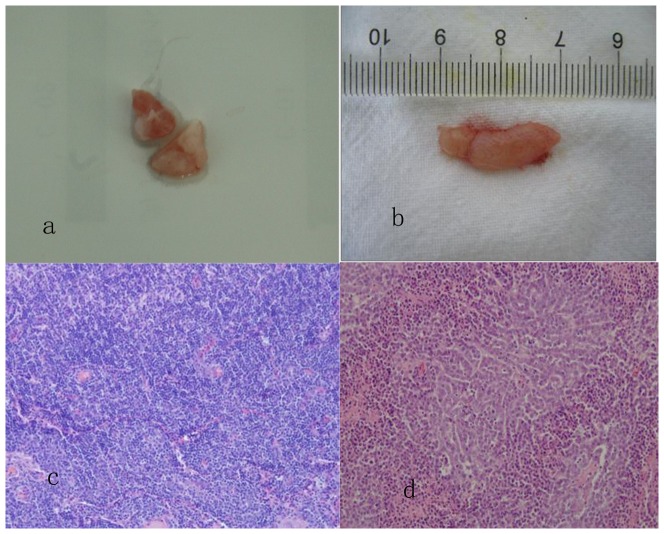
Dissected deep neck lymph nodes of a rabbit. **b**. Measurement of the long diameter of the lymph node. **c**. A reactive proliferation of lymphocytes. (HE×200) **d**. Lymph node metastasis of poorly differentiated squamous cell carcinoma. (HE×200).

Fifty-four lymph nodes were dissected from 10 rabbits. Twenty-six of the 54 lymph nodes were confirmed to be tumor metastases. Five lymph nodes had focal necrosis. (Table 1, Fig 1a, b, d)

**Table 1:** The long and short diameters of lymph nodes in the tumor group.

	Long diameters (mm)	Short diameters (mm)
	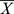 ±S range	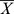 ±S range
**Benign nodes (n = 28)**	10±2.8 5–8	4.8±1.6 3–12
**Malignant nodes (n = 26)**	13.6±3.5 7–25	6.8±2.3 4–17

#### 1.2 MRI Scan

**Figure 2:**
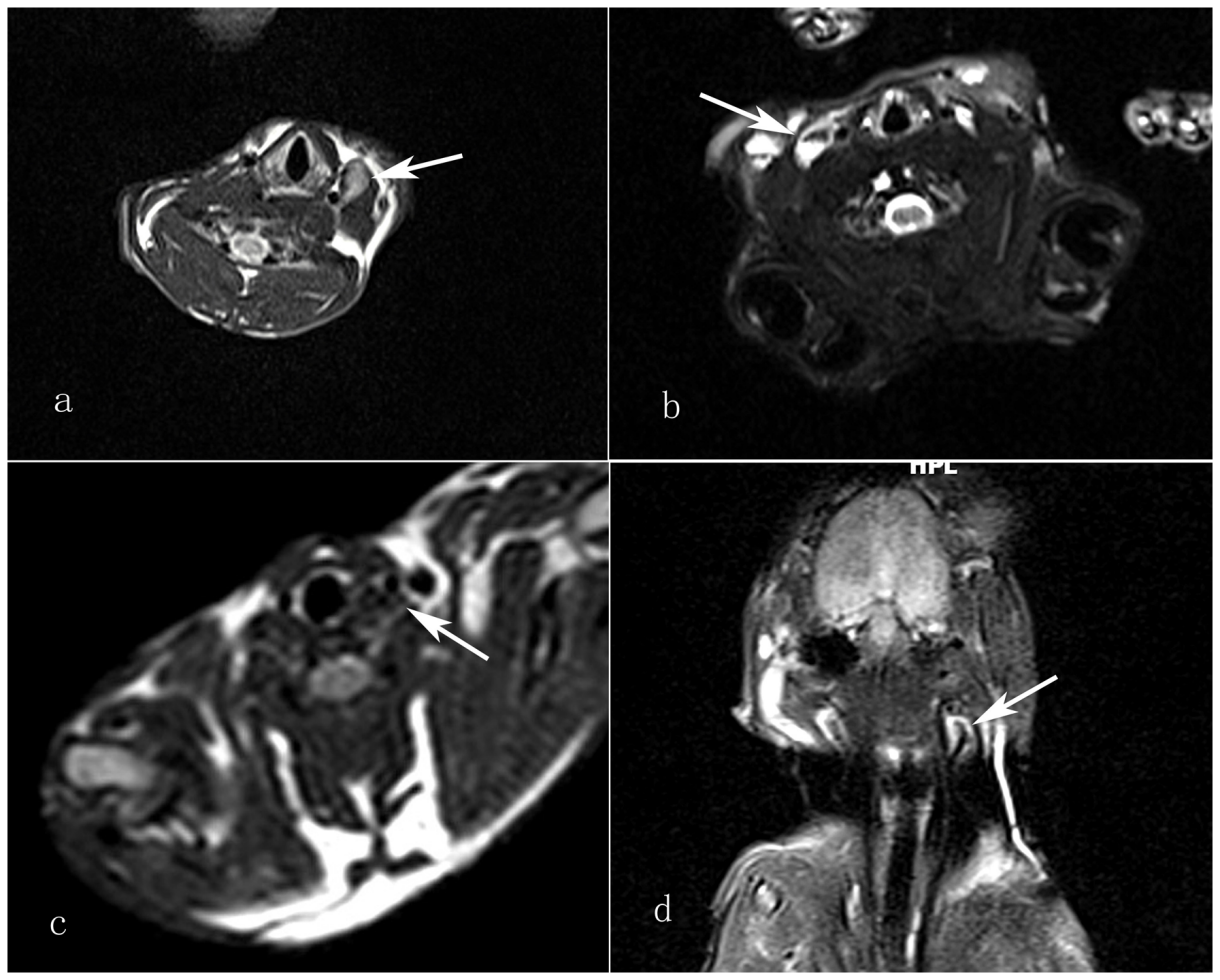
T2-weighted image of the neck shows a metastatic lymph node with partial high signal intensity, significantly higher than that of muscle tissue. (white arrow) **b**. T2-weighted image of the neck shows the metastatic lymph nodes that had irregular signal reduced intensity (white arrow). **c**. The inflammatory lymph nodes showed homogeneous signal falls on T2-weighted image. (white arrow) **d**. The metastatic lymph nodes also had irregular signal reduced intensity on coronal view (white arrow).

The T1 and T2-weighted images of the neck showed that the lymph nodes had partial high signal intensity, and that was significantly higher than that of the muscle tissue. The T2 value was 299.96±49.55 (Table 2, Fig 2a). The necrotic area had a significantly high T2 signal. Twenty of the 54 lymph nodes were diagnosed as tumor metastases according to the images. There were 5 false positives and 17 false negatives. The sensitivity and specificity of the MRI scan were 57.6% and 60.7% (Table 3).

**Table 2:** T2 value before and after injection USPIO.

	T2SI_PRE_	T2SI_POST_	T2_SI (%)_
**Tumor group**	299.96±49.55	107.22±49.55	65
**Inflammatory group**	324.81±49.26	70.92±31.13	79

**P<0.05 compared with before and after injection USPIO.**

**Table 3:** The sensitivity and specificity of USPIO enhanced scan &MRI scan in tumor group.

Method	Sensitivity	Specificity
**MRI scan**	57.6% (15/26)	60.7% (17/28)
**USPIO enhanced scan**	96.1% (25/26)	85.7% (24/28)

**P<0.05 compared with before and after injection USPIO.**

#### 1.3 USPIO Enhanced Scan

Twenty-nine of 54 lymph nodes were diagnosed as tumor metastasis according to the images, while the other 25 nodes were not. Among them, 4 nodes were the false positive and 1 nodes were the false negative. The sensitivity and specificity were 96.1% and 85.7%.(Table 3) On T2-weighted image of the neck showed the metastatic lymph nodes that had no signal reduced intensity or irregular signal reduced intensity. T2 value was 107.22±49.55. (Table 2, Fig 2b, d) The benign lymph nodes showed homogeneous low signal intensity with irregular ring enhanced area on T2-weighted image.

#### 1.4 Statistical analysis

The sensitivity and specificity values were calculated using a 2×2 contingency table. (Table 3) The sensitivity and specificity of the USPIO enhanced scan were significantly higher than the MRI scan. (P<0.05)

### 2. Inflammatory model group

#### 2.1 Histopathologic results

Forty-two lymph nodes were dissected from 10 rabbits. All lymph nodes were found to be reactive hyperplasia. (Table 1, Fig 1c)

#### 2.2 MRI Scan

The T1-weighted image of the neck showed that the lymph node had iso-signal intensity, and the T2-weighted image showed a slightly high signal intensity or mixed high signal intensity. The T2 value was 324.81±49.26 (Table 2).

#### 2.3 USPIO Enhanced Scan

Six lymph nodes were mistakenly categorized as mestastatic nodes and showed multiple patchy signal falls on the T2-weighted image. The other lymph nodes all showed homogeneous signal falls on the T2-weighted image. The T2 value was 70.92±31.13 (Table 2, Fig 2c).The specificity of the USPIO enhanced scan was 85.7%.

## Discussion

Several studies have shown that indirect lymphography can significantly enhance the sensitivity and specificity of MRI for lymph node metastatic diagnosis [14]. Bellin, et al [15] detected lymph node metastases in 30 patients with prostate cancer using ferumoxtran-10. The sensitivity and specificity of that contrast agent were 80% and 100%, respectively. Another study of 80 patients with prostate cancer showed that the sensitivity and specificity of diagnosis increased from 35.4% to 90.5% and 90.4% to 97.8%, respectively, when using USPIO enhanced MRI instead of conventional MRI [16]. In this study, we evaluated the use of USPIO as an MRI contrast agent for the diagnosis of lymph node metastasis of pyriform sinus cancer. The results showed that compared to conventional MRI, the sensitivity increased from 57.6% to 96.1% and the specificity also increased from 60.7% to 85.7%.

In addition, indirect MR lymphography can be used to identify sentinel lymph nodes. Torchia [17] reported the detection of sentinel lymph nodes of pig tongue carcinomas using indirect MR lymphography. Rogers [18] confirmed that rat axillary lymph nodes can easily be seen after a subcutaneous injection of USPIO. In our study, we used a rabbit animal model. Since the distribution of rabbit cervical lymph nodes is similar to that of humans [19], this is a good model for lymph node metastasis research. In the rabbit model of auricular and tongue VX2 carcinoma, the SLN are parotid lymph nodes [20], [21]. Our previous study indicated that pyriform sinus carcinoma metastasizes primarily in the deep cervical lymph nodes [22]. By analyzing of the USPIO-enhanced MR image, we found that the deep cervical lymph nodes were the first group of lymph nodes to develop metastases. These results are consistent with those of our previous study. Visibly, USPIO MR imaging can be used to locate and diagnose the SLN of pyriform sinus carcinoma.

However, we also encountered several issues while using USPIO MR imaging. One was that there is no uniform regulation for the dose of the USPIO contrast agent used in animal experiments; doses ranging from 30 umol to 1 mmolFe/kg have been reported [23]–[25]. Also, doses for intravenous and subcutaneous injection are different. Some studies reported that the lowest dose for subcutaneous injection was 0.028 mg/kg, while the lowest dose for intravenous injection was 0.16 mg/kg [26]. In this study, USPIO was diluted to 1 mg per kg for sub-mucosal injection. And the results confirmed that the quality of the images could meet the diagnostic requirements for lymph node metastasis.

Another issue was the scanning time selection. Tauptiz [27] reported that scanning at 12 h after subcutaneous injection of USPIO generates the best images and scanning at 24 h after intravenous injection reveals the most lymph phagocytes and the optimum enhanced imaging phase [26]. In the preliminary experiment for this study, we performed the MRI scans at 1 h, 2 h, 4 h, 6 h, 12 h, 18 h and 24 h after USPIO injection. We lastly found that scanning at 12 h after USPIO injection generated the best image. The third issue was determining the best MRI examination sequence. The USPIO particle accumulated in cell clusters, and, through the magnetic sensitivity effect, this generated an ultra-small magnetic field gradient at the local site. The transverse relaxation time was shortened by phase proton spin diffusion and irreversible loss. The signal intensity on the T2-weighted images was significantly reduced, and this could be considered negative reinforcement. However, the affection of the T1-weighted images was weak. Therefore, the T2-weighted images of the USPIO enhanced MRI were usually used to diagnose the tumor metastasis.

Currently, the diagnosis of lymph node metastasis with CT scan and MR scan is based on morphological changes. If the short diameter of the lymph node is greater than 15 mm or the ratio of length and short diameter (L/S) is less than 2, the lymph node can be considered metastatic. However, the accuracy of these measures is not high, and is limited for the diagnosis of occult metastasis [28]–[30]. It was reported [31]–[34] that USPIO enhanced MR scanning could reach a minimum threshold of 2 mm in the diagnosis of metastasis, and micro-metastases could be found in normal sized lymph nodes. In this study, the USPIO-enhanced MRI had a higher sensitivity, specificity, and accuracy for the diagnosis of lymph node metastasis than the MRI scan. In the tumor group, one false negative lymph node (by MRI scan) was found to be metastatic by USPIO enhanced MR scanning. The lack of the phagocytic cells in the germinal centers of the lymph nodes could be the main reason for that false negative result [35]. Furthermore, the lower spatial resolution of MRI may also lead to false negative results [36]. In the experiment, some false positive lymph nodes were found in the inflammatory model group. We propose that the results of the image contrast enhancement may have been affected by the time of the USPIO enhanced scanning, an unreasonable choice of slice thickness, a reactive lymphatic hyperplasia, etc. Also, an inadequate dose of the USPIO contrast agent could also have led to false positive results [37]. In addition, in cases of a reactive lymphatic hyperplasia, the germinal centers expanded but lacked of phagocytic cells, and the phagocytes instead gathered in the main medullary sinuses. At this time, the USPIO magnetization effect was concentrated in a small area of the central lymph nodes. The T2 signal declined slightly. All of those factors led to the false positive results [38].

In summary, USPIO-enhanced MRI scanning has some advantages for the diagnosis of cervical lymph node metastases, but it also has some limitations, such as tedious scanning steps and side effects. However, compared with MRI scanning, it has higher accuracy for the diagnosis of metastatic lymph nodes and, thus, is a new direction for the clinical diagnosis of lymph node metastasis.

## Conclusion

Conventional MRI evaluation has some limitations. USPIO-enhanced MRI scanning provides a new direction for the diagnosis of metastatic lymph nodes and has high specificity and sensitivity. Thus, it has important application values in the diagnosis of head and neck cancer lymph node metastasis.
